# A Beginner’s Introduction to Skin Stem Cells and Wound Healing

**DOI:** 10.3390/ijms222011030

**Published:** 2021-10-13

**Authors:** Daniel Díaz-García, Alžbeta Filipová, Idalia Garza-Veloz, Margarita L. Martinez-Fierro

**Affiliations:** 1 Biomedical Research Center, University Hospital Hradec Králové, Sokolská 581, 500 05 Hradec Králové, Czech Republic; daniel.diazg@gmail.com; 2 Department of Pharmacology, Faculty of Medicine in Hradec Králové, Charles University in Prague, Šimkova 870, 500 03 Hradec Králové, Czech Republic; 3 Department of Radiobiology, Faculty of Military Health Sciences in Hradec Králové, University of Defense, Třebešská 1575, 500 01 Hradec Králové, Czech Republic; alzbeta.filipova@unob.cz; 4 Molecular Medicine Laboratory, Academic Unit of Human Medicine and Health Sciences, Universidad Autónoma de Zacatecas, Zacatecas 98160, Mexico; idaliagv@uaz.edu.mx

**Keywords:** skin stem cells, non-healing wounds, skin regeneration, tissue engineering, wound healing

## Abstract

The primary function of the skin is that of a physical barrier against the environment and diverse pathogens; therefore, its integrity is essential for survival. Skin regeneration depends on multiple stem cell compartments within the epidermis, which, despite their different transcriptional and proliferative capacity, as well as different anatomical location, fall under the general term of skin stem cells (SSCs). Skin wounds can normally heal without problem; however, some diseases or extensive damage may delay or prevent healing. Non-healing wounds represent a serious and life-threatening scenario that may require advanced therapeutic strategies. In this regard, increased focus has been directed at SSCs and their role in wound healing, although emerging therapeutical approaches are considering the use of other stem cells instead, such as mesenchymal stem cells (MSCs). Given its extensive and broad nature, this review supplies newcomers with an introduction to SSCs, wound healing, and therapeutic strategies for skin regeneration, thus familiarizing the reader with the subject in preparation for future in depth reading.

## 1. Introduction

Covering an average surface area of 1.85 m^2^, and accounting for ~15% of total body weight, the skin is considered the largest organ in the human body. Its primary function is that of a physical barrier against microbial pathogens, toxic agents, UV light, and mechanical injury [1]. However, this function can also extend into other vital functions, such as thermoregulation, protection against dehydration, and the excretion of waste metabolites [2]. Moreover, the skin also represents a major metabolic site, yielding a broad range of biomolecules, e.g., vitamin D [3].

The skin is composed of two main layers, i.e., the epidermis and the dermis. Previously, another layer had been described within the skin, i.e., hypodermis [4]; however, there is an ongoing controversy in this regard and the hypodermis is now considered as part of the dermis. The skin contains accessories, such as hair, nails, and sweat, and sebaceous glands [5]. In addition, the skin is also populated by nerve receptors that can be triggered by external stimuli (e.g., touch, heat, pain, and pressure) [6]. The skin layers have different thickness according to their anatomical location; for example, the epidermis can be very thin in the eyelids (0.1 mm) whereas it can be thicker in the palms and soles of the feet (1.5 mm). In contrast, the dermis can be ~30–40 times thicker in the dorsal area than the corresponding epidermal layer [2].

**Figure 1:**
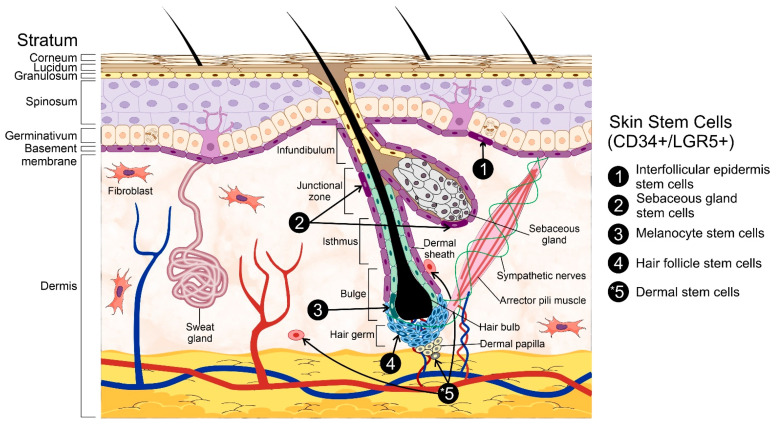
General structure of the skin. The skin is composed of two primary layers: the epidermis and the dermis. The epidermis, hair follicles, and the dermis are the primary skin stem cells reservoirs. Among the different populations of stem cells are hair follicle stem cells, interfollicular epidermis stem cells, sebaceous gland stem cells, melanocyte stem cells, and dermal stem cells. * The dermis represents a larger adult stem cell reservoir than the hair follicle and epidermis put together. Three representative stem cell subpopulations from the dermis (dermal stem cells) including neural crest stem cells, mesenchymal stem cells-like dermal stem cells, and dermal hematopoietic cells are represented in the figure. Some elements of this figure were taken from the Mind the Graph platform, available at www.mindthegraph.com (accessed on 27 September 2021).

The epidermis can be further sub-divided into strata with a unique cell composition, i.e., keratinocytes, dendritic cells, melanocytes, Merkel’s cells, and Langerhans’ cells. These epidermal layers are known as stratum germinativum, stratum spinosum, stratum granulosum, stratum lucidum, and stratum corneum. The first of these strata, also known as the basal cell layer, conforms the inner-most part of the epidermis [2,7]. It is in this layer that different populations of stem cells (SCs) are located, and which, through extensive proliferation and differentiation, provide the great regeneration capacity of the skin and enable the generation of auxiliary structures, e.g., nails and sweat glands [8]. It must be mentioned that the basal cell layer is not the only stem cell niche within the skin as these cells can also be found within the hair follicle (HF), interfollicular epidermis (IFE), and sebaceous glands [8], all of which are contained within the basal layer itself. The stem cells within the skin are usually named after the niche in which they reside in, i.e., hair follicle stem cells (HFSCs), melanocyte stem cells (MeSCs), interfollicular epidermis stem cells (IFESCs), and dermal stem cells (DSCs). Regardless of their niche, these cells are collectively known as skin stem cells (SSCs) (Figure 1).

The main task of these SSCs is to replace, restore, and regenerate the epidermal cells that may have been lost, damaged, or have become pathologically dysfunctional [9,10]. For such end, a carefully orchestrated cell division, both symmetrical and asymmetrical, is required to both maintain the stem cell pool and produce lineage-committed cell precursors [11]. Initially, SSCs were thought to be age-resistant, mostly because their number does not seem to dwindle through time [12,13]. However, despite their longevity, SSCs eventually become unstable or dysfunctional and display a lower differentiation and self-renewal capacity [14].

As previously mentioned, SSCs are found in diverse niches within the skin, of which the hair follicle has been the most studied. The distinct anatomical zones of the HF can house different stem cell types, such as HFSCs and MSCs [15,16]. The bulge region of the HF contains different stem cell populations; however, the exact identity of these cells is still unclear. Regardless, the presence of both proliferative (CD34+/LGR5+) and quiescent (CD34+/LGR5−) stem cells has been described in previous research [16,17].

Overall, the diverse subpopulations of SSCs have specific characteristics that set them apart from one another. For instance, HFSCs are mostly quiescent until triggered by several factors secreted by their progeny and by adjacent dermal cells [18]. Regarding the former, their isolation has been so far complicated by the lack of specific markers to identify them [19]. In addition to the hair follicle bulge, SSCs can also populate the sebaceous glands; however, these stem cells are thought to be unipotent and dedicated exclusively to the renewal of the sebocytes’ pool [16,20]. Other proposed niches are found within the compartments of the dermal papilla (DP) and the dermal sheath (DS) [9,16] and, unlike the stem cells located in the sebaceous gland, those located in both the DP and DS display a greater differentiation capacity, even being able to differentiate into cells of hematopoietic lineages [9], and have also been involved in the maintenance and repair of the dermal tissue. Melanocyte stem cells (MeSCs) are also located in the bulge and hair germ of the HF. Interestingly, their proliferation and differentiation seem to be closely tied to that of HFSCs [21]. Therefore, the concurrent activation of both MeSCs and HFSCs by the signals originating from the latter is hardly surprising. Due to their embryonic origin (i.e., neural crest), MeSCs possess high proliferative and multipotent capacity, which makes them interesting for regenerative medicine [22] and stem cell-based therapies [15,23]. In this regard, dermal stem cells (DSCs) are also considered as an accessible and abundant source for stem cell-based therapies [24] as they display great plasticity and the potential to differentiate into cells of ectodermal, mesenchymal, and endodermal lineages [24,25]. Consistently, the niche of these cells has been localized to the DP and DS [26]. IFESCs, on the other hand, are difficult to isolate and identify due to their unclear location within the basal layer. Therefore, their study has been mostly conducted through indirect means, such as screening with cell surface markers [27,28] or lineage analysis and tissue regeneration assays [29].

Before delving further and in trying to bring greater clarity to the previous paragraph, let us recapitulate the existing models for skin stem cells that are currently being considered. The earliest model describing the hierarchy of stem cells in the interfollicular epidermis suggests the columnar arrangement of keratinocytes stacked in what is known as epidermal proliferative units (EPU) [30]. According to this model, stem cell clones are similar in size and their number remains rather constant during homeostasis. Relatively few basal cells have stem cell properties and can create transit amplifying (TA) cells, which constitute the majority of basal cells. This model suggests that TA cells go through several proliferation cycles before leaving the basal cell layer and follow their terminal differentiation program [31].

Despite the seeming adequacy of this model, a relatively recent study showed that the size of epidermal clones increases over time, which contradicts the previous EPU model. Therefore, a stochastic model was proposed where the basal cells have inherent progenitor characteristics and their differentiation occurs at random. This apparent asymmetry in the cell population results in a scaling behavior in clone size and distribution. Thus, according to this model cell clones become fewer in number and have variable size [32]. Further, this model proposes the existence of a quiescent stem cell population with as few as four to six divisions per year and where progenitor cells present a balanced, although still random, differentiation pattern. However, one in five mitotic cycles would result in progenitor loss, thus suggesting that the population of both stem cells and progenitors might be heterogeneous and with different degree of competence [33].

The validity of these theories was later tested in a mathematical simulation in which both the classical hierarchical model and the stochastic model described above would result in stem cell depletion [34]. Therefore, a third model proposed the existence of both a quiescent stem cell population and a committed progenitor population with stochastic differentiation fate [35]. Interestingly, this model could also explain the diminished healing capacity observed in the later stages of life, as the number of stem cells would decline with age. However, it must be kept in mind that all of these models are based in murine models and are not fully applicable in humans. Thereby, further research in this regard is still needed. Due to the extensive nature of this subject in particular, we suggest an excellent review by Dr. Helena Zomer et al. providing greater detail and context [36].

Due to the extensive and complex nature of the subject, the present review conveys a broad overview on SSCs, wound healing and the signaling pathways involved therein, as well as some of the current strategies in stem-cell based treatment strategies for wound healing.

## 2. Skin Stem Cells and Wound Healing

### 2.1. Cell Signaling Pathways and SSCs

The health and maintenance of the skin is tightly regulated through the secretion of diverse cytokines, chemokines, growth factors, and the activation of specific signaling pathways [37,38]. Mitogens such as insulin-like growth factor (IGFs), fibroblast growth factor 7 (FGF-7), FGF-10, and the epidermal growth factor receptor (EGFR) facilitate epidermal regeneration [39]. Several signaling pathways have been linked to the development and regeneration of the skin; however, none of them seem as relevant or essential as Wnt, which regulates cell proliferation, differentiation, migration, and polarity [40,41]. Somewhat unique—the Wnt signaling pathway can be activated through diverse means, one of which is known as the canonical Wnt/β-catenin pathway. This canonical pathway is typically active during tissue morphogenesis, thus regulating its development [42,43]. Interestingly, Wnt signaling drives skin development and maintenance through both canonical and non-canonical signaling cascades [42,43]. So far, 19 Wnt genes have been identified in the human genome [42]. These proteins are secreted into the extracellular environment and bind to the frizzled (Fz) family of receptors (e.g., lipoprotein receptor-related proteins 5 and 6 [LRP-5/6], receptor tyrosine kinase like orphan receptor 2 [ROR2], and receptor like tyrosine kinase [RYK]) to activate various signaling pathways [44,45]. These Wnt receptors can be blocked by different proteins, e.g., Dickkopf protein (Dkk), secreted frizzled-related protein (SFRP), or Wnt inhibitory factor (WIF), which regulate the activation of the signaling cascade [46,47]. On the other hand, Wnt signaling can also be modulated by R-spondin and leucine-rich repeat-containing G-protein coupled receptor proteins [48,49].

The early skin tissue displays a dynamic crosstalk between the epidermis and the dermis during embryonic development that drive the formation of the basement membrane, the stratification of the epidermis, and formation of the HF [50]. The differentiation of ectodermal cells into epidermal cells is also controlled by Wnt signaling, which inhibits the function of fibroblast growth factors (FGFs) in the ectodermal cells, thus promoting the expression of bone morphogenetic proteins (BMPs) and committing these cells towards an epidermal fate [51]. These cells differentiate into K7-expressing cells, i.e., keratinocytes, and form the basal layer in the embryonic epidermis. The keratinocytes in the basal layer switch from K8/K18 expression to K5/K14 [52]. During early epidermal stratification, the epidermal basal cells create the periderm which isolate the basal cells [53]. The intermediate layer is then created between the basal layer and the periderm [54]. The cells located in this layer proliferate and mature into spinous cells expressing K1/K10; these cells in turn give raise to involucrin-positive granular cells, which differentiate into filaggrin- and loricrin-expressing cornified cells [55]. The latter forms the cornified envelop in the surface of the skin and functions as a barrier against the environment. Once the stratification of the skin is complete, the epidermis is now constituted by an inner layer of basal cells with high proliferation capacity as well as of supra-basal layers of differentiated cells.

The HF, as a major stem cell niche within the skin, has been strongly associated with Wnt signaling during its induction [56]. HF morphogenesis occurs in three main steps: the formation of the hair placode, HF organogenesis, and cell differentiation [57]. These events are tightly regulated by Wnt, thus reaffirming its role as a master regulator. As a first step, dermal fibroblasts, stimulated by Wnt, induce the aggregation of epidermal basal cells, thus forming hair placodes. Thereafter, this placode express Wnt ligands that induce the formation of a dermal condensate [58]. At the same time, the placode keeps growing and produces an invagination into the dermis that joins with the dermal condensate to form the primary hair germ (HG), the first structure of HF organogenesis. The epidermal cells penetrating the forming dermis generate the hair peg while the dermal condensate transforms into a dermal papilla (DP). The bottom section of the hair peg becomes thickened and forms the hair bulb, which encloses the DP. The differentiated epidermal cells within the hair peg will create the hair shaft. Cell differentiation starts once the developing HF reaches the subcutis. At this stage, the DP becomes thinner and completely enclosed, whereas the sebaceous gland starts its development in the upper HF. After this, a hair shaft raises from the skin surface and the HF reaches its maximal length [59]. As previously mentioned, SSCs occupy multiple niches within the skin, including the basal layer of the interfollicular epidermis (IFE) and the bulge region of the HF [60]. Consistently, Wnt also plays an essential role in the maintenance, activation, and differentiation of the SSCs located in these niches.

HF stem cells are split into two sub-populations, i.e., one residing in the bulge (hair follicle stem cells, HFSCs) and another localized in the secondary hair germ (sHG) [61]. Bulge HFSCs remain mostly quiescent; on the other hand, sHG stem cells are active during HF formation [62]. In contrast, the epidermis is continuously renewed by the basal cells in the IFE, whose pool remains constant thanks to their capacity for asymmetric division [63]. In this regard, the hierarchical and stochastic models suggest how the cells in the basal layer of the IFE can be replenished [64]. The former states the existence of slow-cycling SSCs within the proliferative units of the IFE, thus generating short-lived transit-amplifying cells (TACs) that later become differentiated. The stochastic model, on the other hand, proposes that the progenitor cells in the basal layer of the IFE have the same potential to create progenitors or differentiate into supra-basal cells [65]. Due to their proliferative and multipotential capacity, SSCs are essential for skin regeneration and wound healing [66]. Within the IFE, SSC populations can be classified as label-retaining cells (LRCs) and non-LRCs during their phenotyping. It must be mentioned that these subpopulations differ at the molecular level and commit into different lineages; regardless, they are functionally similar and are capable of replenishing the opposing niche upon injury [67]. Interestingly, the maintenance of the IFE does not require a clear SSC or TAC hierarchy [68].

### 2.2. Principles of Wound Healing and Skin Regeneration

**Figure 2:**
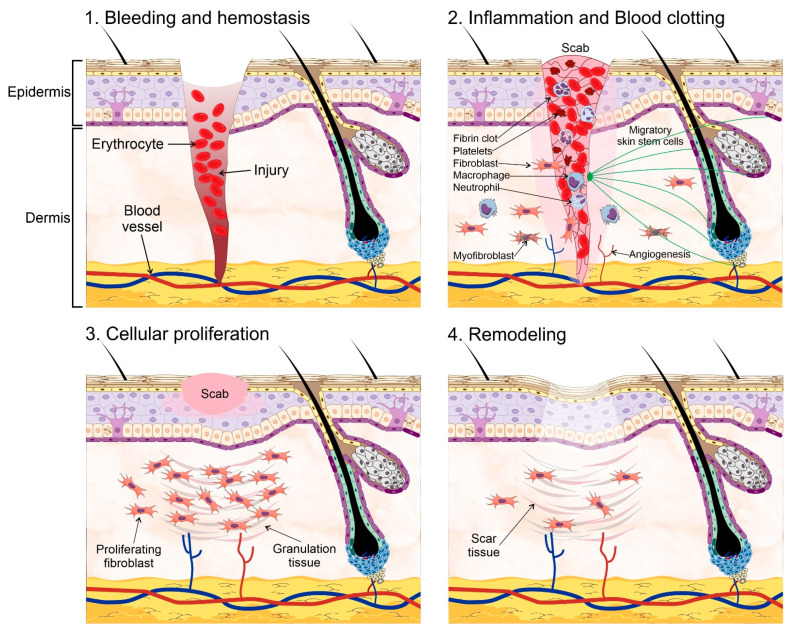
Wound healing process. Wound healing and skin regeneration. Wound healing is a highly conserved mechanism that includes processes such as: (**1**) bleeding and hemostasis, in which blood flow slows and a clot forms to prevent blood loss during an injury. (**2**) Inflammation and blood clotting, in which the wound becomes sealed by fibrin which forms a temporary matrix occupied by immune cells whose task is to remove dead tissue and control infection, fibroblasts are then recruited into the site of the injury and promote angiogenesis and the recruitment of fibroblast-derived myofibroblasts, which contract the wound area. Skin stem cells are mobilized into the site of injury at this stage to begin the process of re-epithelialization starting from the edge of the wound. (**3**) Cellular proliferation, in which the recruited fibroblasts secrete collagen, and form granulation tissue, a scab is formed on the site of injury. (**4**) Extracellular matrix (ECM) remodeling, in which new ECM components are secreted by both fibroblasts and epidermal keratinocytes, which remodel the matrix through the expression of matrix metalloproteinases. Some elements of this figure were taken from the Mind the Graph platform, available at www.mindthegraph.com (accessed on 27 September 2021).

The skin is an essential component in the protection against environmental hazards, e.g., UV light, pathogenic agents, and dehydration. However, the continuous exposure to such threats also compromises its integrity. Therefore, wound healing and skin regeneration are indispensable for the health and survival of higher organisms. Wound healing is a highly conserved mechanism among species, and includes processes such as inflammation, blood clotting, cellular proliferation, and extracellular matrix (ECM) remodeling [69,70]. During the inflammatory phase, the wound becomes sealed by fibrin, which forms a temporary matrix occupied by immune cells whose task is to remove dead tissue and control infection. Fibroblasts are recruited afterwards into the site of the injury and secrete collagen, form granulation tissue, and promote angiogenesis and the recruitment of fibroblast-derived myofibroblasts, which contract the wound area. SSCs are mobilized into the site of injury at this stage to begin the process of re-epithelialization starting from the edge of the wound. Finally, new ECM components are secreted by both fibroblasts and epidermal keratinocytes, which also remodel the matrix through the expression of matrix metalloproteinases (MMPs). Amazingly, the regenerated skin tissue is able to regain ~80% of its normal strength in as little as 3 to 4 months (Figure 2).

Despite the conserved nature of wound healing, its results differ between species. Some organisms are able to completely regenerate their skin (e.g., zebrafish, axolotl, and toads), including the formation of secretory appendages [70,71] or the pigmentation pattern of their skin [70], thus making this new skin indistinguishable from the original [69]. In mammals, however, wound healing often results in the formation of scar tissue that lacks any of the original appendages (i.e., hair follicles, nails, and glands). Despite its apparent shortcomings, this new scar tissue can still meet the basic functions of the skin regarding protection against pathogens and dehydration. Notwithstanding its inherent survival advantage, this process does not mitigate the drawbacks of extensive scarring on the quality of life of the afflicted individual. The ability to fully restore the skin to its original state has been highly sought after, with extensive research dedicated toward this goal throughout the years.

Skin maintenance depends upon the proliferation and differentiation capacity of the basal layer of the epidermis, which gives raise to suprabasal cells, the granular layer, and finally to the stratum corneum. After injury, the process of re-epithelialization should occur as soon as possible to prevent the loss of the barrier function, which includes protection against dehydration or death from a pathogenic infection. Therefore, the rapid migration and proliferation of epithelial cells is critical to close the site of injury and restore the normal function of the skin, a process that is mainly mediated by skin stem cells residing in the basal layer of the skin.

Wound healing begins with the recruitment of basal cell subpopulations expressing keratin 14 and involucrin into the wound area, the former of which have greater proliferation and differentiation potential and, thus, are able to survive for longer in comparison with the latter [35]. In addition to basal layer cells, there are specific progenitor cells within the hair follicle, particularly in the bulge, upper bulge, sebaceous gland junction, and infundibulum, which are able to differentiate into epidermal cells and, thus, contribute to skin regeneration [72]. Keratin15 expressing SSCs within the bulge/secondary hair germ region of the hair follicle can migrate toward the central region of a wound after full epidermal excision [73,74]. Interestingly, the expression of keratin15 is not necessary for normal epidermal homeostasis, suggesting that its expression occurs only in response to injury. Once K15þ SSCs have migrated to the epidermis, they adopt an epidermal phenotype, disappearing thereafter. Suggesting that these HF bulge stem cells are mainly involved during the acute phase of injury. However, it must be kept in mind that extensive injury can deplete the cell populations responsible for skin regeneration, severely compromising the re-epithelialization process or make it outright impossible.

Other SSCs subpopulations express the marker Lrig1. These cells are mostly located within the junctional zone between the hair follicle bulge, sebaceous gland and infundibulum [75]. These cells are able to differentiate into all epidermal lineages in mice [75]. After suffering an injury, Lrig1 cells migrate into the wound area and remain in situ for up to 1 year, thus contributing toward skin regeneration in a semi-permanent manner [76]. In addition, cells expressing the marker Lgr6, located within the isthmus area, also contribute to re-epithelialization [77]. These cells are able to reconstitute every epithelial lineage of the skin and to support the formation of hair follicles when co-transplanted with inductive dermal cells. These progenitor cells can regress into self-renewing SSCs in response to injury and, interestingly, they can reside within other compartments in addition to the HF bulge (e.g., isthmus and junctional zone). The HF bulge can also contribute other cell populations that participate in the healing process, one of which expresses Gli1. Gli1þ cells are mostly found in the upper bulge area of the HF and contribute toward wound healing by establishing long-term progenitors after injury; however, these cells are highly dependent upon the perineural stem cell niche to maintain their ability to differentiate into epidermal cell lineages after injury [78]. Despite its contribution, the HF is not necessary for skin regeneration, although it does facilitate and accelerates this process [79]. Interestingly, wound healing is greatly influenced by the morphogenesis stage of the HF, being faster during anaphase [80], when the HF is producing angiogenic factors [81]. Table 1 shows the different skin SC populations according to their location and progenitor markers.

### 2.3. Scarless Wound Healing

The formation of fibrotic scar tissue can be an unavoidable consequence of wound healing and, sometimes, it can result in severe cosmetic and psychological consequences. Normally, acute wound repair occurs only in response to severe injury; interestingly, this process is not directly driven by the SSCs present in the HF bulge. Instead, short-lived transient amplifying cells (TACs) are created for the healing process [73]. Only slow-cycling stem cells and committed progenitor cells residing within the interfollicular epidermis (IFE) have a substantial contribution toward the repair and long-term regeneration of the damaged tissue [35]. As previously mentioned, there are different and highly heterogenous stem cell pools in the skin, each with a specific role during wound healing. For instance, dermal papillae (DP) possess a mesenchymal cell population associated with HF growth and development, thereby constituting an important reservoir of multipotent stem cells [88]. Other cells, designated as skin-derived precursors (SKPs), also contribute to skin maintenance, wound healing, and HF morphogenesis. Due to their self-renewing and multipotent capacity, these cells are considered as SSCs [89].

While the role of SSCs in wound healing cannot be understated, relatively recent research has demonstrated the essential role of dermal fibroblasts in wound healing and scarring. These fibroblasts are usually classified into two main lineages according to their localization in either the upper or lower dermis, the former of which contributes to re-epithelization and hair follicle formation, whereas that the latter synthesizes the fibrillar extracellular matrix (ECM) in the reticular dermis and are heavily involved in dermal repair after injury, infiltrating the wound bed during healing. These fibroblasts express α-smooth muscle actin (α-SMA), thus presenting a myofibroblastic phenotype [90]. These fibroblast lineages have a different response to Wnt/β-catenin signaling and, instead, Hedgehog (Hh) signaling promotes the proliferation of papillary fibroblasts whereas that TGF-β regulates ECM remodeling in the reticular dermis [91]. It must be noted that two different pools of reticular fibroblasts, Sca1+/CD26+ and CD26+/En1+, constitute the majority of all dermal fibroblasts and are responsible for the connective tissue deposition leading to scar formation [91,92].

Curiously, scar formation is absent during the early gestational period of mammals [93]. The mechanisms involved are currently unknown; however, it may be possible that the existent differences between the developing skin and that of an adult, such as the decreased tensile strength of the early fetal skin, could play a role in this process. In this regard, the focal adhesion kinase (FAK) has been linked with fibrosis through the expression of extracellular-related kinase (ERK), which induces the secretion of monocyte chemoattractant protein-1 (MCP-1), a protein associated with diverse fibrotic disorders [94,95]. Interestingly, the inhibition of the inflammatory FAK-ERK-MCP-1 pathway attenuates scar formation [94]. The scar-free healing process observed in fetal tissues has been strongly associated with a reduced inflammatory response; in contrast, the healing of pathologically compromised injuries often involves an unregulated inflammatory process [96]. Therefore, it may be surmised that the inclusion of anti-inflammatory agents could push the wound healing process toward a regenerative pathway instead [97]. In this regard, the fibroblast growth factor 9 (Fgf9) can modulate the inflammatory response [98] and promote the regeneration of the HF, which are normally lost when scarring occurs [99]. The administration of interleukin 10 (IL-10) also contributes toward the regeneration of normal skin structures and attenuates the expression of pro-inflammatory molecules [100]. On the other hand, the inhibition of the chemokine receptor type 4 (CXCR-4) prevents the recruitment of pro-inflammatory cells towards the wounded area and the activation of fibroblasts, thus resulting in scarless healing [101]. Additional studies have identified diverse cell populations that contribute toward the formation of scars. One of such populations express disintegrin and metalloproteinase domain-containing protein 12 (ADAM12), both of which are involved in fibrosis and scarring [102]. En1-derived fibroblasts have also been involved in cutaneous scarring as they are major contributors of connective tissue during scar formation [92]. Myofibroblasts play a key role in normal wound healing by promoting the contraction of the wound and ECM production [93]. Myofibroblasts are naturally heterogeneous and their population is known to include fibroblasts, mesenchymal stem cells (MSCs), smooth muscle cells, endothelial cells, and fibrocytes [103]. Lastly, adipocytes have also been found during wound healing, which, apparently, are required for the recruitment of fibroblasts into the wounded area [104].

Unequivocally, the regeneration capacity of the skin is driven by stem cells; therefore, stem cell-based therapies for wound healing are mostly aimed at recalcitrant wounds, although their capacity for scarless healing is still debated. The ability to regulate the wound environment is key for stem cell-based therapies, as they can downregulate the inflammatory response [105,106,107]. Regrettably, the extensive application of stem cells in wound healing therapy is restricted by practical limitations [108]. Regardless, other strategies seem promising; one of such involves the use of umbilical cord (UC)-MSC conditioned media, which can induce dermal fibroblasts to behave like fetal fibroblasts, e.g., lower myofibroblast differentiation, diminished TGF-β1/TGF-β3 ratio, and increased ECM remodeling. Unsurprisingly, skin injuries treated with UC-MSC conditioned media heal faster and accumulate less collagen [109]. Other studies with MSC derived from human amniotic fluid have shown great potential to inhibit the pro-fibrotic activity of TGF-β1 as well as the ability to reverse the phenotype of myofibroblasts back into a fibroblast-like state.

Wound healing has been linked to great extent with Wnt signaling, of which the canonical pathway (Wnt/β-catenin) plays a prominent role in skin regeneration and repair. Since this pathway has already been extensively described in other reports, we will only provide a brief recapitulation of its involvement in wound healing. The levels of β-catenin are remarkably high during normal wound healing with scar formation [110] and other studies have shown a clear association between TGF-β and the Wnt/β-catenin pathway during fibrosis, e.g., hypertrophic scars and keloids [111]. Moreover, human keloids display an upregulated Wnt-3a expression, inducing the transition of endothelial fibroblasts to mesenchymal cells, and thus to the accumulation of collagen [112]. On the other hand, TGF-β signaling is crucial for a pro-fibrotic fibroblast phenotype, which also involves the strong participation of the Wnt pathway. In the absence of β-catenin, TGF-β1 cannot promote hyperplastic wounds in mice [113]. TGF-β also downregulates the Wnt-antagonist Dickkopf-1 (Dkk-1), thereby upregulating the Wnt/β-catenin signaling pathway [114]. The application of Wnt pathway inhibitors leads to the formation of rete pegs and dermal papillae after wound closure, which would be otherwise lacking under normal healing conditions with scar formation. Regarding the latter, its formation can also be prevented by inhibiting the Wnt pathway [115]. Its apparent efficiency notwithstanding, a careful balance must be struck in this regard as the inhibition of β-catenin can also lead to decreased contraction, migration, and collagen production, thus having an overall negative effect on wound healing [116]. On the other hand, the activation of epidermal Wnt/β-catenin signaling can promote the expansion of upper dermal fibroblasts enabling HF growth, which is typically absent under normal wound healing conditions [90,93]. Therefore, upper dermal fibroblasts are currently considered as a plausible alternative in the promotion of scarless wound healing [117].

### 2.4. Chronic Wounds

The process of wound healing can sometimes be impaired or compromised, as is the case of type 1 and 2 diabetic patients. Recent years have seen a dramatic increase in the number of cases of chronic non-healing wounds, thus becoming a serious public health issue worldwide [118]. Non-healing wounds represent a life-threatening complication and often precede limb amputation in diabetic patients [119]. In addition, chronic wounds can also deplete the local SSCs populations due to their frequent cycling during the healing process [120]. It must be mentioned that the normal wound healing steps, such as inflammation and MMP expression, can also prevent correct ECM remodeling and, thus, the normal progression of the wound healing process.

As we already know, wound healing is a complex and multi-step procedure that requires an inflammatory reaction, wound clotting, re-epithelialization, remodeling regulation, and stem cell control. In recent years, the role of SSCs in wound healing has been recognized as an integral part in the healing of diabetic wounds, as they are essential during the steps of wound closure and tissue remodeling. Therefore, the direct loss of SSCs, their impaired migration capacity, and/or faulty differentiation capacity may have a profoundly negative effect on wound healing. One of such impairing effects has been directly related with telomere stability, as it can hinder both the proliferation and migration capacity of SSCs [121]. The telomeres have been involved in a wide range of cellular processes, including ageing and cancer. Moreover, telomere maintenance is crucial for the longevity of stem cells. The upkeep of the telomeres’ length is responsibility of an enzyme known as telomerase, which is normally downregulated in somatic cells but highly expressed in both cancer and stem cells. An integral part of this enzyme is known as telomerase reverse transcriptase (TERT) [122]. TERT is involved in some types of trauma, particularly through the activation of NF-κB and autophagy [123]. In SSCs, however, TERT expression is required for normal cell proliferation and migration; further, its expression is closely associated with the activation of the Wnt/β-catenin signaling pathway [124]. The inactivation of this signaling pathway, which has been associated with the inflammatory response in diabetic ulcers, wound proliferation and remodeling [125], has also been linked with the expression of telomerase; although the precise mechanism still remains obscure.

To understand the intricacies of chronic wounds, we must delve a bit deeper into the process of wound healing. As it is well known, collagen is a major component of the skin’s extra cellular matrix (ECM), providing it with a sound structural integrity by forming a scaffold for cell adhesion. There are several types of collagens, of which the skin mostly contains types I and III, with the former providing tensile stiffness, whereas the latter has greater distensible characteristics. It might be due to this reason that collagen type III plays a crucial role in the early phases of wound healing [126]. During healing, collagen provides adherence sites for the activated platelets, facilitating their clumping to stop bleeding. In addition, collagen also provides support and connection points for the fibroblasts recruited into the site of injury, thus promoting wound contraction [127,128]. In diabetic patients, however, collagen metabolism is altered, delaying the process of re-epithelialization and impairing the migration and proliferation of keratinocytes and fibroblasts, thereby compromising the wound healing process as a whole.

The multiple, and complex, steps of wound healing also include the participation of several cell types, cytokines, and extracellular matrix components [129]. In addition, angiogenesis is also an essential part of the process and its malfunction is a known feature of diabetic wounds [130]. Further, dysfunctional vascular endothelial cells also contribute to the poor wound healing capacity of diabetic patients [130]. In this regard, previous studies have reported the enhanced healing and angiogenesis of diabetic wounds induced by transplanted mesenchymal stem cells (MSCs) [131,132,133]; thus, suggesting that the recovery of a faulty vascular endothelial cell function is dependent upon these stem cells, although the exact mechanisms by which this happens is poorly understood. Regardless, it is suggested that MSCs might stimulate cell survival and functional recovery within the site of injury or that perhaps they regulate the local microenvironment and immune response [134].

### 2.5. Wound Healing Therapy

Cultured epidermal cell sheets (CES) have been used in the treatment of non-healing wounds with relative success and, while they do indeed help to save lives, the normal function and appearance of the skin after the transplant is still in need of improvement. Therefore, several synthetic skin substitutes have been developed to enhance the function, strength, and integration capacity of the transplanted CES. In this regard, it was discovered that enriching these CES with SSCs results in a better outcome regarding function [135], scar formation [136], and long-term regeneration of the skin [137], and it has been proven that CES transplants in severely burned areas increase patient survival [138,139]. To provide further insight into this fascinating issue, we highly recommend the work of Jang at al. [140] and Jackson et al. [141] as additional reading material. Regardless, there are still serious issues, such as engraftment deficiency, appearance, and function, which remain to be solved [142]. Other therapeutic approaches involve the use of synthetic skin substitutes consisting of a matrix that can be seeded with cells; however, these allografts are not without limitations, including faulty engraftment [143]. Other limitations include their simplistic structure, which is unable to recreate the native conditions needed for cell signaling [144]. Newer skin substitutes are formed by composite layers compatible with the ECM, several cell types, and ECM-bound growth factors [145]. Enriching these skin substitutes and CES with SSCs before transplantation may have an improved outcome, including the successful engraftment of the cultured SSCs [137]. However, the variable number of SSCs per patient, along with non-standardized protocols for their culture and phenotype preservation, have become important factors in the failure of these transplants. Therefore, the use of integrin β1-compatible substrates that can also mimic the SC niche is being implemented to improve the clinical outcome of these therapies.

Despite the great advances made in this regard, recent years have seen an increasing interest over stem cell-based regenerative therapy instead, mostly due to their self-renewing and multi-potential capacity [146]. Therefore, it is not surprising that Mesenchymal stem cells (MSCs) are being considered in therapeutic wound healing strategies, mostly because they can be easily obtained, isolated, and expanded in vitro [147]; in addition, MSCs display immunomodulatory, reparative, and regenerative characteristics through paracrine signaling [148]. Their differentiation potential and ability to induce the regeneration of the damaged epithelium through the secretion of growth factors, inflammatory cytokines, and chemo-attractants makes them especially attractive for wound healing therapy [149]. The use of MSCs’ conditioned media increases the healing rate of skin injuries in a significant manner in terms of re-epithelization, neovascularization, collagen deposition, and collagen arrangement [150]. A closer analysis revealed that this improved healing was due to the cytokines, microvesicles and exosomes secreted by the MSCs into the media, all of which influence cell migration and promote a well-organized set of molecular and cellular events necessary for wound healing [151].

It must be mentioned that there are endogenous cutaneous MSCs, most of which are located within the dermal papilla, the dermal sheath, and in the interfollicular dermis [152,153]. MSCs can also be activated upon injury and recruited into the wound from the adjacent adipose tissue; interestingly, bone marrow MSCs (BM-MSCs) are also recruited into a wound during the early inflammation phase, remaining in the healed tissue afterwards [154,155,156,157]. Although there is a limited number of studies, MSCs have been successfully applied in chronic wounds [158], acute surgical wounds and diabetic ulcer wounds [159]. The use of MSCs in wound healing results in greater tensile strength in the wound [160], reduced scarring [161], lower wound contraction [162], and upregulated collagen expression [163]. Moreover, the anti-inflammatory effect of MSCs reduces scarring [164], probably due to rapid wound closure, improved angiogenesis, and collagen deposition induced by the paracrine signaling of MSCs [165].

The paracrine activity of MSCs can be attributed to their exosomes, which are secreted into the micro-environment [166] and contain a broad range of signaling molecules, including growth factors, cytokines, miRNAs, and chemokines [167]. These MSC-exosomes can induce tissue regeneration, restore tissue homeostasis, and accelerate wound healing [168]. The advantage of using MSC-exosomes instead of their source is that the former can fuse directly with the target cells, they can be stored and transported at −70 °C, their clinical use is easy to standardize and control, and there is no risk of engraftment failure or tumorigenesis [169]. Moreover, MSC-exosomes can ameliorate the inflammatory response in the injury by promoting the switch of the recipient’s macrophages to an anti-inflammatory M2 phenotype [170]; in addition, MSC-exosomes exert a strong immunosuppressive effect by regulating the activation of B- and T-lymphocytes [171,172]. MSC-exosomes can also drive angiogenesis and downregulate the expression of the matrix metalloproteinase (MMP)-9 [173], whose high expression has been related to poor wound healing [174]. MSC exosomes can also regulate the proliferation and migration of fibroblasts, thus participating in the formation of granulation tissue and collagen synthesis [175,176], which can be advantageous in the treatment of chronic diabetic wounds [177]. Finally, MSC exosomes regulate ECM remodeling by driving the synthesis of elastin, and collagen type I and III [178], therefore expediting wound healing.

## 3. Conclusions

This review describes the composition of the skin, SSCs, the cellular and molecular principles involved in wound healing, as well as some therapeutic strategies for skin regeneration. Despite the extensive research conducted so far on this subject, much remains to be explored and, undoubtedly, further efforts in this regard will provide greater clarity in the development of therapeutic strategies for chronic wounds and extensive scarring. As an example, although not strictly within the realm of stem cells, some of the innovations in this regard include the use of gas plasma technology to promote wound healing through increased oxygenation and vascularization of the wound tissue, induced death of senescent cells, redox signaling, and antiseptic activity [179]. Another novel approach consists in the delivery of a fibroblast growth factor 2 (FGF-2) coding plasmid enveloped in cationic lipid nanoparticles (cLP), which can promote cell proliferation and migration in as little as 48 h after treatment. Although this has only been tested in vitro so far, it might be a promising therapeutic strategy regardless if its results can be validated in vivo [180]. As a final suggestion, we highly recommend the reviews by Branski et al. [181], explaining the use of gene therapy in wound healing, and by Rasouli et al. [182], who highlight the importance of the extracellular matrix (ECM) and its potential use as a clinical tool in wound healing therapy. We, the authors, wish the readers good luck in their future research endeavors and hope this review has provided a friendly approach in the world of skin stem cells and wound healing.

## Figures and Tables

**Table 1:** Skin stem cells and their identifying cell surface markers.

Location	Stem Cell Population	Progenitor Markers	Reference
Basement membrane	Interfollicular epidermis stem cells	CD34, LGR5	[82]
Junctional zone of hair follicle	Sebaceous gland stem cells	Lrig1, CD34, LGR5	[78,83]
Isthmus zone of hair follicle	Dermal stem cells	Lgr6, Plet1, Gli1, Lrig1, MTS24, K15^low^	[78,83]
Bulge zone of hair follicle	Melanocyte stem cells	Krt15, Lgr5, CD34, Sox9, Lhx2, Tcf3, Nfatc1, Gli1	[78,83]
Hair germ zone of hair follicle	Hair follicle stem cells	Krt15, CD200, Gli1, Lgr5, Sox9, CD34	[78,83]
Dermal papilla zone of hair follicle, dermal sheath	Neural crest stem cells	Nestin, fibronectin, vimentin, versican, musashi, Sox9, Sox2, Slug, Snail, Twist, Pax3, CDw90, SH2, SH4, CD105, CD166, CD44, CD49 d-e, HLA class I, p75, Sox10, Oct4, NGFRP75	[84,85]
Dermal papilla zone of hair follicle, connective tissues of dermis, reticular dermis, hypodermis	Mesenchymal stem Cells, such as dermal stem cells	CD90, CD44, CD59, ICAM-1, VCAM-1, vimentin, CD34, CD133, ABCB5, SSEA-3, OCT4, Sox2, Nanog, Par-4	[84,86]
Dermal papilla zone of hair follicle, dermal sheath	Dermal hematopoietic cells	CD90, CD44, CD59, ICAM-1, VCAM-1, vimentin, CD45, CD34, OCT4, CD117, Sca-1	[84,87]

## Data Availability

No new data were created or analyzed in this study. Data sharing is not applicable to this article.
